# Effect of color protection treatment on the browning and enzyme activity of *Lentinus edodes* during processing

**DOI:** 10.1002/fsn3.2895

**Published:** 2022-04-20

**Authors:** Tong Lin, Zhiguo Zhou, Chunmiao Xing, Jiahui Zhou, Gongjian Fan, Chunyan Xie

**Affiliations:** ^1^ 71219 College of Life Science Langfang Normal University Langfang China; ^2^ Technical Innovation Center for Utilization of Edible and Medicinal Fungi in Hebei Province Langfang China; ^3^ Edible and Medicinal Fungi Research and Development Center of Hebei Universities Langfang China; ^4^ 74584 College of Light Industry and Food Engineering Nanjing Forestry University Nanjing China

**Keywords:** blanching, browning, color protection, *Lentinus edodes*, oxidase

## Abstract

Fresh *Lentinus edodes* (*L. edodes*) are prone to browning (including enzymatic and nonenzymatic browning), which affects their quality and leads to economic losses during later processing. This study explored various effective color protection methods (color protection reagent and/or blanching) for inhibiting the browning of *L. edodes*. First, a single‐factor experiment and a response surface method were used to optimize the concentration of the color retention reagent. The compound color retention reagent (comprising 0.1% phytic acid, 0.8% sodium citrate, and 0.5% *d*‐sodium erythorbate) had the smallest total color difference (ΔE) value, suggesting that the compound color reagent had a better inhibiting effect than the single agent. Following this, the blanching conditions were studied; the polyphenol oxidase (PPO) activity was the lowest when the blanching temperature was 90°C and blanching time 180 s, indicating that browning is likely to be minimal. Finally, comparing the oxidase activity and total color difference (ΔE) revealed that combining the two color protection methods inhibits browning better than using a single method (color protection reagent or blanching). In addition, the polysaccharide and vitamin C (VC) contents of *L. edodes* under optimal color protection conditions were determined, which were 0.96 and 2.54 g/100 g fresh weight (FW), respectively. The results demonstrated that this color protection method effectively inhibits browning, reduces the nutritional loss, and improves the quality of *L. edodes*.

## Graphical abstract

The effect of a composite color retention reagent was better than that of a single reagent. Blanching can effectively inhibit the activity of polyphenol oxidase (PPO) of *Lentinus* *edodes*. Using a compound color protectant in addition to blanching may effectively inhibit the browning and reduce the nutrient loss of *L. edodes*.

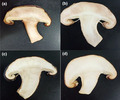

## 1 INTRODUCTION

*Lentinus edodes* (*L. edodes*), cultivated in Asian countries for thousands of years, are among the most popular edible mushrooms. Their strong odor makes them preferable to consumers (Chen et al., 2018; Sheng et al., 2021; Silva et al., 2007). With the rapid development of industrialized cultivation techniques, *L. edodes* have been cultivated in Asia, Europe, North America, and Australia (Liu, Yuan, et al., 2013). *L. edodes* contain approximately 60% carbohydrates, 20% proteins, 10% fiber, 5% lipids, 5% ash, and various vitamins and minerals, making them a high protein and low‐fat food (Hu et al., 2020; Morales et al., 2019; Xu et al., 2014). *L. edodes* are the second most popular edible fungi globally, largely because of their taste, therapeutic value, and nutritional benefits (Huang et al., 2019; Sheng et al., 2021; Ziaja‐Sołtys et al., 2020). However, fresh *L. edodes* can easily deteriorate in terms of browning after harvesting.

Fresh *L. edodes* have a short storage time and shelf life owing to their high enzymatic activity and water content (Chen, Lv, et al., 2020). The browning reactions specifically limit the shelf life to within a few days, with polyphenol oxidase (PPO) and peroxidase (POD) being the primary enzymes responsible for the enzymatic browning process (Singh et al., 2018). Here, PPO catalyzes the oxidation of the OH functional group on the carbon atom of the monohydroxyphenol benzene ring to *o*‐dihydroxyphenol and further catalyzes the dehydrogenation to *o*‐quinone, with the oxidation of the phenolic compounds into quinones resulting in the browning of *L. edodes* tissues. POD is another important enzyme that causes tissue browning using H_2_O_2_ as a catalyst for the oxidation of the phenolic compounds (Xu et al., 2019). This enzymatic browning, which is caused by the oxidation of phenolic substances and formation of quinones and their polymers under aerobic conditions through the action of oxidases (POD and PPO) (Sikora et al., 2020; Wang, Ye, et al., 2021; Zheng et al., 2019), naturally reduces mushroom quality. Furthermore, nonenzymatic browning, specifically the Maillard reaction or the caramelization that mainly depends on reducing the products’ sugar content (Corzo‐Martınez et al., 2012), is another factor that causes the browning of mushrooms during processing (Xiao et al., 2017), such as cooking, frying, drying, and storage. Based on these findings, several strategies have been developed to inhibit the browning of *L. edodes*. For instance, the use of intermittent ozone treatment and biodegradable phase change materials can maintain the color of *L. edodes* during storage at the low temperature (Kong et al., 2021; Liu et al., 2020). *Oudemansiella radicata* polysaccharide coatings decrease phenolic oxidation, reduce browning, and inhibit ripening and senescence of *L. edodes* at 4℃ (Liu et al., 2021). These approaches for inhibiting the browning of *L. edodes* essentially focus on the low‐temperature storage. However, few studies have focused on an approach to restrain the browning of *L. edodes* during processing under non‐low‐temperature conditions.

Various color protection reagents, including sodium ascorbate, citric acid, and ethylenediaminetetraacetic acid (EDTA), have been used to inhibit browning and control spoilage during fruit or vegetable processing (Carocho et al., 2018; Liu, Zou, et al., 2013). During processing, the color change (e.g., browning) of mushrooms is an important index for selecting the color protection reagents. The chemical antibrowning agents are divided into antioxidant, reducing, chelating, and acidifying agents (Singh et al., 2018), with antioxidants being the most commonly used reagents for inhibiting browning because of the reaction with oxygen. Reagents are used to resist oxidation or inhibit oxidase activity. However, the effect of using a single protection reagent is limited, and the development of composite reagents is crucial to addressing the issue of browning.

Thermal blanching is another essential operation used in the processing of various vegetables and fruits. Blanching not only contributes to the inactivation of POD and PPO but also affects other quality factors of the products, including modifying the product texture, preserving the color and nutritional value, removing any trapped air, changing the physical properties, and removing any pesticide or toxic constituents (Bonnechère et al., 2012; Claeys et al., 2011; Wang, Fang, et al., 2021; Zhang et al., 2018, 2020). Several studies have reported that blanching decreases the reducing sugar content of the product, which further inhibits the browning and improves the color of mushrooms (Pimpaporn et al., 2007). Therefore, using a compound color protection reagent in addition to blanching may effectively inhibit browning during the processing of *L. edodes*.

This study aimed to identify the best approach for preventing the browning of *L. edodes* during processing. First, the optimal composite color protector composition was determined using the single‐factor method and response surface method (RSM). The effects of blanching conditions on the browning of *L. edodes* were assessed before determining the efficacy of using the combination of a compound color protection reagent and blanching. In addition, the oxidase activity (PPO and POD) and reducing sugar and vitamin C (VC) contents under different color protection conditions were also analyzed, which further demonstrated the efficacy of the method. Overall, this study provides a theoretical basis for preventing the browning of mushrooms during processing and can be used as a reference for relevant future research.

## 2 MATERIALS AND METHODS

### 2.1 Materials and chemicals

Fresh *L. edodes* with similar maturity, color, and size were purchased from Langfang Yuanchen Supermarket. All other chemicals and reagents were obtained from Sinopharm Chemical Reagent Co., Ltd. Deionized water was used throughout all the experiments.

### 2.2 Treatment of *L. edodes* using color protection reagents

*Lentinus edodes* were gently washed with water to remove any foreign materials adhered to their surface. Various samples were cut into approximate 5‐mm flakes and soaked in different color protection reagents for 30 min; the control group samples were soaked in deionized water. After soaking, all samples were allowed to stand for 2 h at room temperature; the color was assessed at 0 and 2 h. Before the assessment, the water on the sample surface was removed using a water‐absorbent paper.

All samples were placed flat on a tray and assessed using an NR110 colorimeter (Shenzhen Sanenchi Technology Co., Ltd.); the following factors were measured: L* (brightness), a* (red + and green−), and b* (yellow + and blue−). The total color difference (ΔE) was then calculated using a method reported by Criado et al. (2021).

### 2.3 Blanching treatments

The cleaned *L. edodes* were cut into nearly 5‐mm slices and then soaked in aqueous solutions at different temperatures (75, 80, 85, 90, 95, and 100℃) for different periods (90, 120, 150, 180, and 210 s). Following this, the samples were placed in deionized water and left to cool for 3 min before determining the enzyme activity of the oxidases.

### 2.4 Enzyme assay

For the analysis of the oxidase activities, the samples (*L. edodes* treated using a composite color retention reagent, blanching, or composite color retention reagent with blanching and untreated *L. edodes*) were ground into a paste and extracted using a 0.1 M phosphate buffer solution (pH 7.0) containing 5% (w/v) polyvinylpyrrolidone (Islam et al., 2014). The mixtures were then centrifuged at 12,000 *g* at 4°C for 20 min, and the supernatants were used as the crude enzyme extract for the PPO and POD.

The PPO activity was assessed using the spectrophotometric method described by Mirshekari et al. (2019). In brief, 1 ml of the supernatant was mixed with 2 ml of a buffered substrate (100 mM sodium phosphate, pH 7.0, and 50 mM pyrocatechol) before the change in absorbance was monitored at 410 nm. One unit (U) of PPO activity was defined as the enzyme amount that caused a 0.01 absorbance increase per minute under the assay conditions. The specific PPO activity was expressed in terms of U/g fresh weight (FW).

The POD activity was also determined spectrophotometrically at 470 nm, as proposed by Jiang et al. (Jiang, 2012). The reaction mixture contained sodium phosphate buffer (50 mM pH 6.0), guaiacol (5 mM), H_2_O_2_ (5 mM), and the supernatant (50 μl). One unit (U) of POD activity was defined in terms of the enzyme amount that resulted in a 0.01/min change in absorbance under the test conditions. The specific POD activity was expressed in terms of U/g FW.

### 2.5 Determination of VC content

The samples were ground into a paste and extracted using a 2% (w/v) metaphosphoric acid solution (Shan et al., 2019). The mixtures were then titrated against a 2,6‐dichloroindophenol solution (Guo et al., 2012), and the results were expressed in mg/100 g FW.

### 2.6 Determination of reducing sugar and crude polysaccharide contents

The reducing sugar and crude polysaccharide contents were extracted according to the methods described by Wang et al. (Wang et al., 2018). Here, the reducing sugar content was determined using 3,5‐dinitrosalicylic acid at 540 nm (Yu et al., 2020). The polysaccharide content was determined using phenol–sulfuric acid and a spectrophotometer at 490 nm (Chen & Huang, 2019).

### 2.7 Experimental design

An optimization experiment was performed via RSM to determine the optimal ratio of the compound color protection reagents, alongside adopting a Box–Behnken design model. The experimental design comprised three factors, with three levels for each factor. The independent variables were the concentrations of phytic acid (0.09%, 0.10%, and 0.11%, A), sodium citrate (0.6%, 0.8%, and 1.0%, B), and *d*‐sodium erythorbate (0.4%, 0.5%, and 0.6%, C; Table S1). The dependent variable was the total color difference (ΔE).

The second‐order polynomial model (Equation 1), which describes the relationship between dependent and independent variables, was used to determine the response data from the central composite design:

(1) $$Y=b_{0}+\sum b_{i}X_{i}+\sum b_{\mathrm{ii}}X_{\mathrm{ii}}^{2}+\sum b_{i\;j}X_{i}X_{j}$$

where *Y* is the response variable; values of *X_i_* (*i* = 1–3) represent the independent variables; *b_0_* is the constant coefficient (also known as the intercept); and *b_i_*, *b_ii_,* and *b_ij_* (*i* and *j* = 3) are the linear, quadratic, and cross‐product regression coefficients, respectively.

### 2.8 Statistical analysis

All statistical analyses were performed in triplicate and reported in terms of means ± standard deviations. The results were processed using the SPSS v23.0 software (Denis, 2018). Statistical significance was evaluated using the analysis of variance (ANOVA) technique, with a difference of *p* < .05 regarded as significant.

## 3 RESULTS AND DISCUSSION

### 3.1 Optimal concentrations of the color protection reagents of *L. edodes*

**FIGURE 1:**
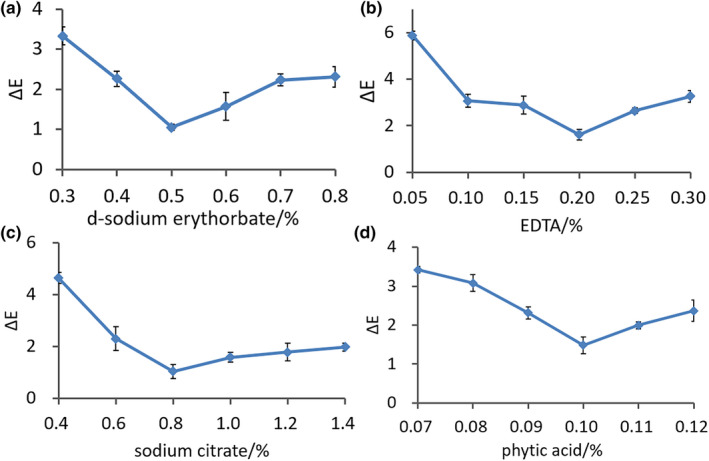
Screening of different concentrations of the color retention reagent. (a) Variation in ΔE at different *d*‐sodium erythorbate concentrations. (b) Variation at different EDTA concentrations. (c) Variation at different sodium citrate concentrations. (d) Variation at different phytic acid concentrations. EDTA, ethylenediaminetetraacetic acid; ΔE, the total color difference

In this study, four chemical reagents (*d*‐sodium erythorbate, EDTA, sodium citrate, and phytic acid) were selected to prevent browning of the *L. edodes*. The total color difference (ΔE), a widely used parameter for determining color differences perceptible by the human eye, of different color protection reagents was then calculated. Erythorbic acid is one of the main antioxidants that has been studied in relation to preventing the browning of fruits or vegetables (Ioannou & Ghoul, 2013). As shown in Figure 1a, the browning of *L. edodes* initially decreased and then increased as the concentration of *d*‐sodium erythorbate increased. The ΔE value was the smallest (1.05 ± 0.08; *p* < .05) when the concentration of *d*‐sodium erythorbate was 0.5%, indicating that this was the optimal concentration for inhibiting the browning.

Sodium citrate and EDTA are antioxidants and chelating agents that can form a complex with PPO or react with its substrates. These compounds potentially form copper (Cu) complexes, where the Cu present in the enzyme structure leads to a reduction in browning (Singh et al., 2018). Phytic acid is also an antioxidant and an acidifying agent that can control browning by decreasing the pH value. PPO is inactivated at a pH below 3.0. As shown in Figure 1d, the ΔE value was 1.48 ± 0.21 (*p* < .05) when the phytic acid content was 0.10%, indicating that this was the optimal concentration for controlling browning. When the content of EDTA and sodium citrate was 0.2% and 0.8%, respectively, the ΔE value was the smallest at 1.62 ± 0.22 and 1.03 ± 0.28 (*p* < .05), respectively (Figure 1b and c).

### 3.2 Optimization of the compound color retention reagent ratio using the RSM

Different chemical antibrowning reagents act on different mechanisms, and combining them may control the enzymatic browning better. The optimal concentration of each color retention reagent was determined through the above‐mentioned experiments described. EDTA had the best effect at a concentration of 0.2%; however, this concentration value exceeds the maximum dose requirement of food additives (75–500 parts per million). Therefore, phytic acid, sodium citrate, and *d*‐sodium erythorbate were selected as the independent variables to form an optimal composite color retention reagent via RSM. Table 1 shows the response values (ΔE) at different experimental combinations for the variables, and Table 2 lists the ANOVA and regression analysis results.

**TABLE 1:** Experimental design and data for optimizing the compound color protection reagent ratio

Runs	A (phytic acid)	B (sodium citrate)	C (*d*‐sodium erythorbate)	ΔE
1	0	0	0	0.94
2	0	−1	−1	2.12
3	0	0	0	1.21
4	0	0	0	0.87
5	−1	1	0	2.84
6	1	0	1	2.68
7	0	−1	1	3.56
8	−1	0	−1	3.53
9	1	1	0	3.64
10	0	1	1	1.76
11	0	0	0	0.92
12	1	0	−1	2.60
13	0	0	0	0.85
14	0	1	−1	3.40
15	−1	0	1	3.27
16	−1	−1	0	3.86
17	1	−1	0	3.02

Abbreviation: ΔE, the total color difference.

**TABLE 2:** Analysis of variance (ANOVA) and regression analysis results for the total color difference ΔE

Source	Sum of squares	*df*	Mean of square	*F* value	*p* Value
Model	19.37	9	2.15	42.3	<.0001*
*A: phytic acid*	0.31	1	0.31	6.02	.0439*
*B: sodium citrate*	0.1	1	0.1	2.05	.1955
*C: d‐sodium erythorbate*	0.02	1	0.02	0.39	.5528
*AB*	0.67	1	0.67	13.13	.0085*
*AC*	0.029	1	0.029	0.57	.4744
*BC*	2.38	1	2.38	46.85	.0002*
*A^2^*	7.63	1	7.63	150.05	<.0001*
*B^2^*	4.53	1	4.53	89.04	<.0001*
*C^2^*	2.15	1	2.15	42.26	.0003*
Residual	0.36	7	0.051		
*Lack of fit*	0.27	3	0.091	4.29	.0966
*Pure error*	0.084	4	0.021		
Cor Total	19.73	16			
*R^2^*		.9819			
Adj *R^2^*		.9587			

Note

*Significant at *p* < .05.

As shown in Table 2, the model was very significant (*p* < .05) and was thus a good fit for data analysis. The *R^2^* and adjusted *R^2^* values were .9819 and .9587, respectively, indicating that the model was suitable for the space design navigation. The lack of fit value was insignificant (*p* > .05), which is desirable for a good model. The linear terms of phytic acid, interactions between phytic acid and sodium citrate and the sodium citrate and the *d*‐sodium erythorbate, and the quadratic terms of the phytic acid, sodium citrate, and *d*‐sodium erythorbate had a significant effect (*p* < .05) on the ΔE. The effects of phytic acid, sodium citrate, and *d*‐sodium erythorbate on the ΔE can be described using the following equation:

$$\begin{array}{c} \Delta E=+0.96‐0.20A‐0.11B‐0.050C+0.41AB+0.085AC‐0.77BC+1.35A^{2}+1.04B^{2}+0.71C^{2} \end{array}$$

**FIGURE 2:**
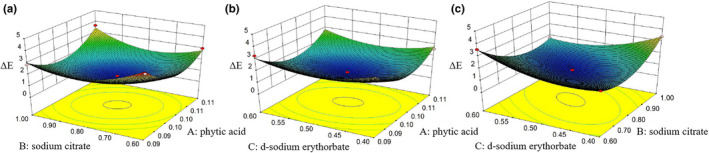
Response surface plot showing the effect of phytic acid, sodium citrate, and *d*‐sodium erythorbate concentrations on the ΔE. (a) 3D surface curve for the effect of phytic acid and sodium citrate concentrations. (b) 3D surface curve for the effect of phytic acid and *d*‐sodium erythorbate concentrations. (c) 3D surface curve for the effect of sodium citrate and *d*‐sodium erythorbate concentrations. 3D, Three‐dimensional; ΔE, the total color difference

Figure 2 and Figure S1 show the ΔE response to the independent variables. There was a significant interactive effect between the three factors (phytic acid, sodium citrate, and *d*‐sodium erythorbate). The browning effect of *L. edodes* was the lowest when the concentrations of phytic acid and sodium citrates were 0.1% and 0.8%, respectively (Figure 2a and Figure S1a). As shown in Figure 2b and Figure S1b, the optimal effect of inhibiting the browning of *L. edodes* was achieved when the concentration of phytic acid and sodium citrate was 0.1% and the concentration of *d*‐sodium erythorbate was 0.5%; however, ΔE was the smallest when the concentration of *d*‐sodium erythorbate was 0.5% and that of sodium citrate was 0.8% (Figure 2c and Figure S1c). The contour plots revealed the effects of phytic acid, sodium citrate, and *d*‐sodium erythorbate concentrations on the ΔE in terms of *L. edodes*. Thus, the best composite color retention reagent composition ratio was as follows: phytic acid = 0.1%, sodium citrate = 0.8%, and *d*‐sodium erythorbate = 0.5%.

### 3.3 Effects of blanching conditions on the browning of *L. edodes*

**FIGURE 3:**
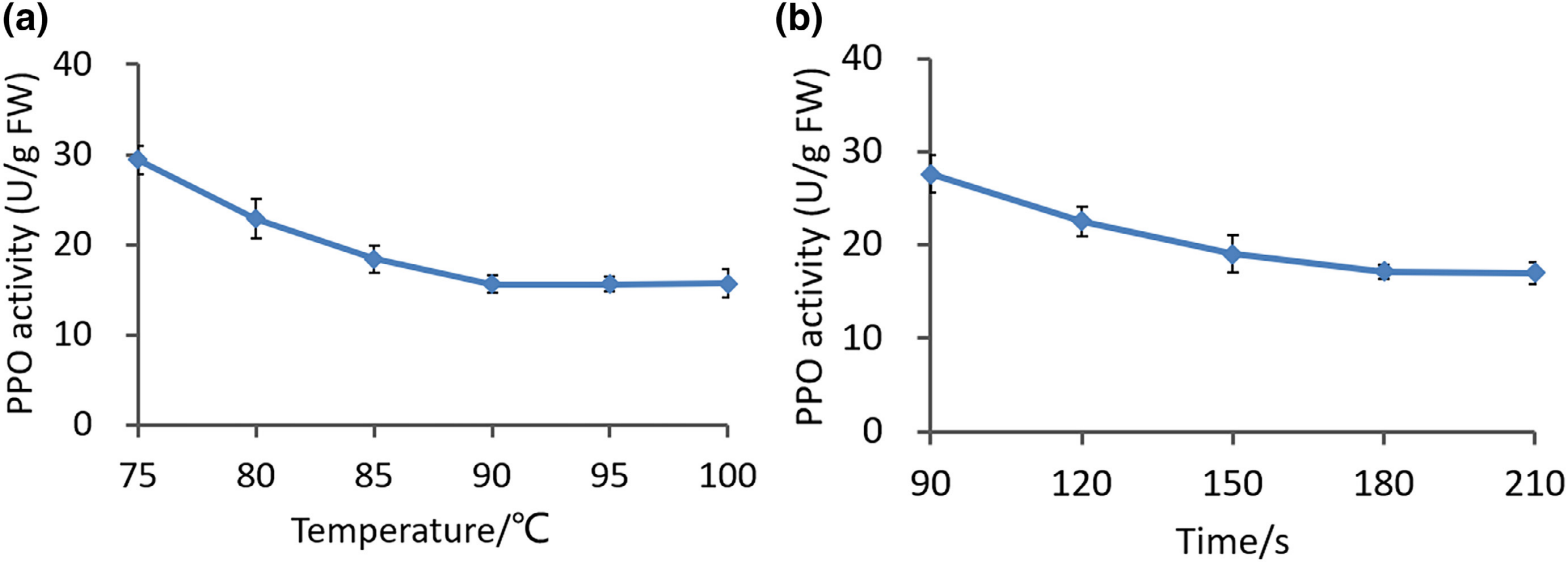
PPO activity under different blanching conditions. (a) PPO activity with different blanching temperatures. (b) PPO activity with different blanching times. PPO, polyphenol oxidase

Postharvest browning, which mainly occurs because of the conversion of phenolic substances into quinones via oxidation, is the limiting factor in the later mushroom processing stages. PPO is the main enzyme that causes the browning of vegetables or fruits (Huang et al., 2017), and blanching effectively inhibits enzyme activity (Xiao et al., 2017). PPO inactivation depends on the heating bath temperature and time, and any loss in quality during the blanching process can be minimized by appropriately selecting the temperature–time schedule. As shown in Figure 3a, the PPO activity gradually decreased with the increase in the temperature. When the temperature exceeded 90°C, the PPO activity remained largely unchanged as the temperature increased; the recorded activities were 15.65 ± 0.92, 15.64 ± 0.78, and 15.72 ± 1.54 U/g FW (*p* < .05) at the blanching temperatures of 90°C, 95°C, and 100°C, respectively. Similarly, when the blanching time increased, the PPO activity gradually decreased before remaining unchanged when the period exceeded 180 s; the recorded PPO activities were 17.15 ± 0.69 and 17.00 ± 1.20 U/g FW (*p* < .05) at the blanching periods of 180 and 210 s, respectively. In terms of minimizing the economic cost during the processing, the best blanching temperature for *L. edodes* is 90°C, whereas the best blanching period is 180 s. Under these conditions, the PPO activity is the lowest, which indicates that browning is likely to be minimal under these conditions.

### 3.4 Inhibiting browning using a combination of compound color protection reagent and blanching

**FIGURE 4:**
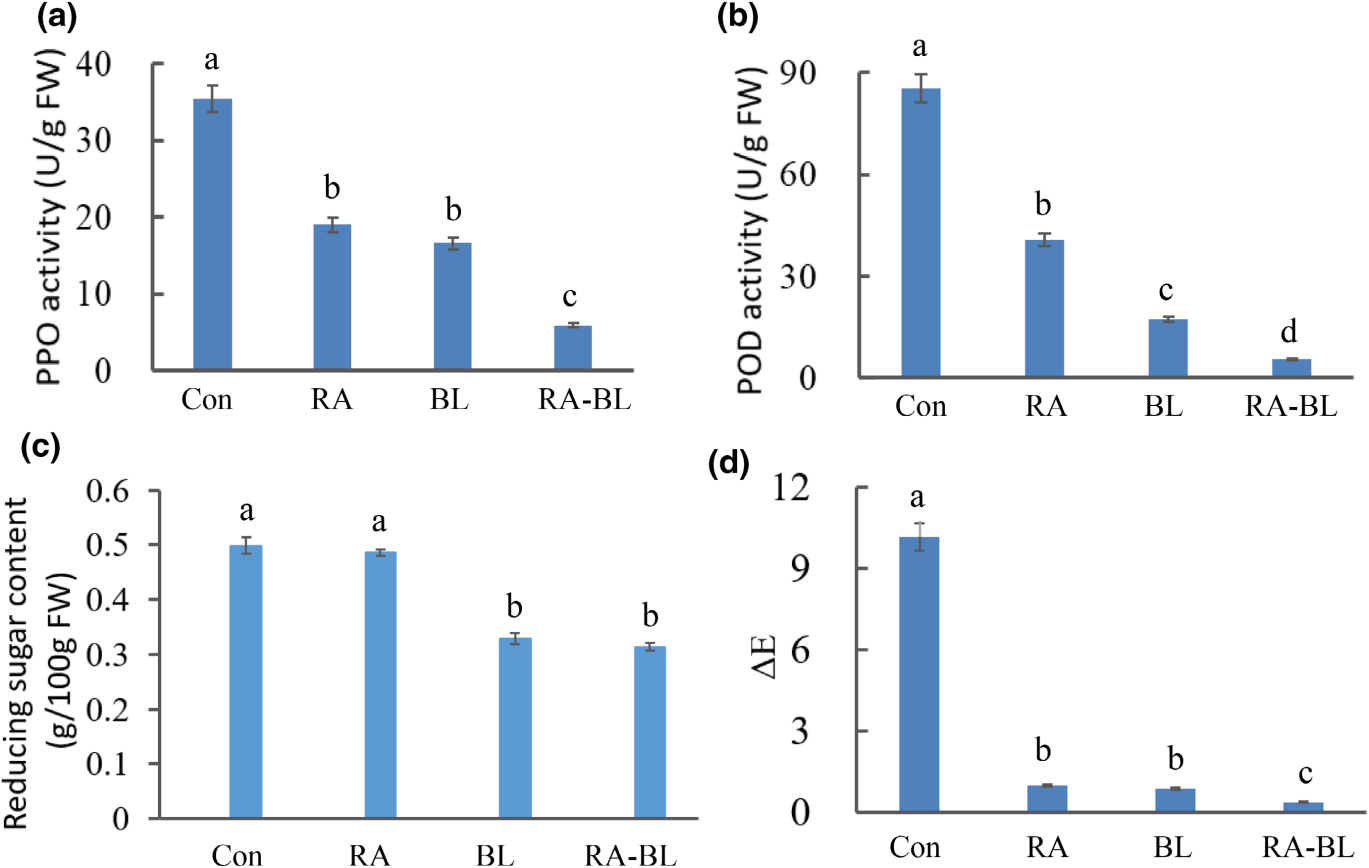
Comparison of oxidase activity and ΔE of *Lentinus* *edodes* treated using different color protection methods. (a) PPO activity with different color protection methods. (b) POD activity with different color protection methods. (c) Reducing sugar content with different color protection methods. (d) ΔE with different color protection methods. BL, *L. edodes* treated with blanching; Con, untreated *L. edodes*; POD, peroxidase; PPO, polyphenol oxidase; RA, *L. edodes* treated with composite color retention reagent; RA–BL, *L. edodes* treated with compound color protection reagent/blanching method; ΔE, the total color difference

Compound color protection and blanching are both important means of inhibiting the browning of *L. edodes*, and the combination of the two is potentially more effective. As shown in Figure 4a, the PPO activity of *L. edodes* treated using a color retention reagent (labeled RA) and blanching (BL) was 19.03 ± 1.04 and 16.66 ± 0.95 U/g FW, respectively, whereas that of the untreated sample (Con) was 35.34 ± 2.02 U/g FW (*p* < .05). The PPO activity was only 5.88 ± 0.22 U/g FW when using a combination of compound color protection reagent and blanching (RA–BL), indicating that the combination of the two results in higher enzymatic browning inhibition. As mentioned above, POD is another important enzyme that causes the enzymatic browning of mushrooms (Wu et al., 2018) as it has a synergistic effect on the formation of the brown polymer with PPO. Several studies have stated that the coordinated action of antioxidant enzymes (PPO and POD) is a major mechanism for the resistance to browning (Laura et al., 2010; Li et al., 2019). The POD activity of Con was 85.35 ± 7.18 U/g FW, whereas those of RA and BL were 40.78 ± 3.61 and 17.33 ± 2.13 U/g FW, respectively (*p* < .05; Figure 4b). When the *L. edodes* samples were treated using the RA–BL combination, the POD activity was only 5.61 ± 0.78 U/g FW, indicating the lowest degree of browning.

In addition to enzymatic browning, nonenzymatic browning is also an issue with *L. edodes* and is mainly related to reducing sugar content. As shown in Figure 4c, the reduced sugar content of *L. edodes* treated using RA was almost the same as that of the Con sample (nearly 0.49 g/100 g FW; *p* < .05). This finding indicated that the use of a color retention agent alone does not effectively inhibit nonenzymatic browning. However, the reducing sugar content of *L. edodes* following BL and RA–BL treatment decreased to approximately 0.31 g/100 g FW, indicating that the nonenzymatic browning is likely to be reduced. Reducing sugar content helps prevent the Maillard reaction or caramelization during processing, thereby inhibiting browning. The ΔE of RA–BL was 0.38 ± 0.03, those of RA and BL were 0.87 ± 0.15 and 0.56 ± 0.09, respectively, and that of Con was 10.16 ± 0.17(*p* < .05; Figure 4d), all of which were consistent with the oxidase (PPO and POD) activities. Overall, the results indicated that although both color retention reagents and blanching can effectively inhibit the browning of *L. edodes*, the treatment using a composite of both is more effective than when using a single color protection method.

### 3.5 Effect of color protection method on the polysaccharide and VC contents of *L. edodes*

**FIGURE 5:**
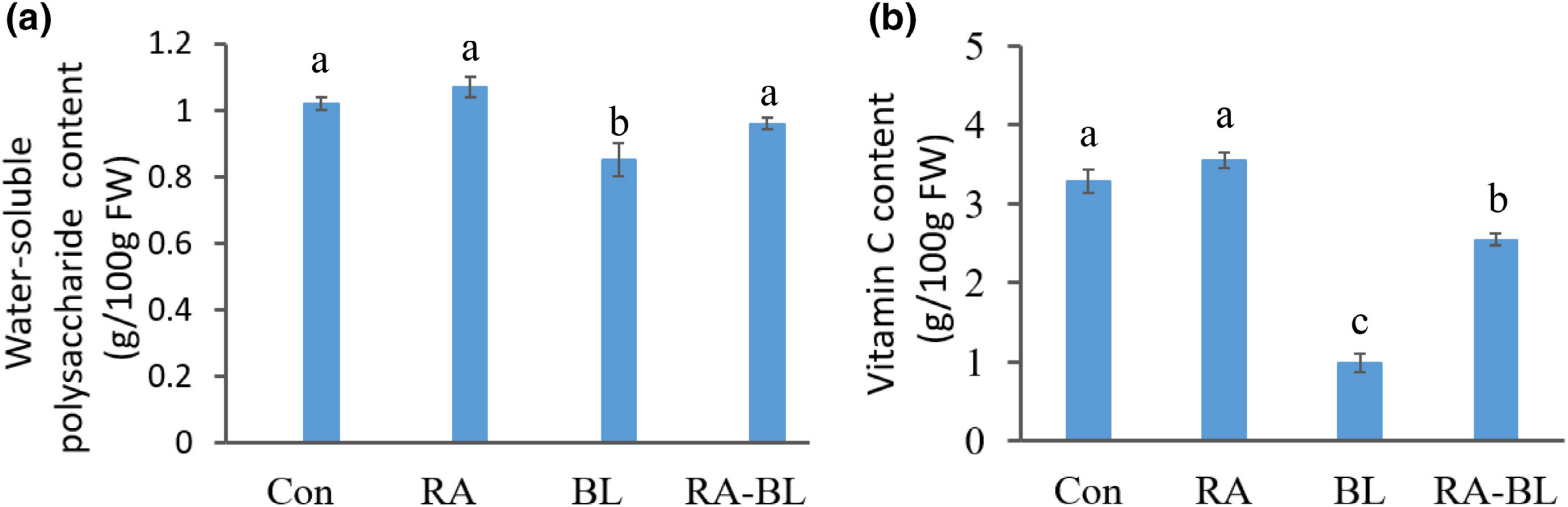
Effect of color protection on the nutritional components of *Lentinus* *edodes*. (a) Water‐soluble polysaccharide (WSP) content with different color protection methods. (b) Vitamin C (VC) content with different color protection methods

*Lentinus edodes* have great nutritional value with great potential for therapeutic application (Chaturvedi et al., 2018; Kala et al., 2021). In recent years, the polysaccharide in *L. edodes* has been widely studied regarding its potent antitumor, anti‐oxidative, and immunomodulating properties (Chen, Liu, et al., 2020; Song et al., 2020; Wang et al., 2019). Reducing the loss of polysaccharide content during processing is beneficial for improving the quality of *L. edodes* and presents a standard for evaluating the color protection methods. As shown in Figure 5a, the ratios of water‐soluble polysaccharides (WSP) isolated from Con and RA were 1.02 ± 0.022 and 1.07 ± 0.031 g/100 g FW (*p* < .05), respectively, indicating that the compound color retention reagent did not affect the WSP content. However, the highest drop in WSP content (0.85 ± 0.046 g/100 g FW) was observed with the BL sample, indicating that using this method will result in some loss in the polysaccharide content. This may be because heating changes the mycelial structure, which facilitates WSP extraction (Sun et al., 2019). There was only a slight decrease in the WSP content (0.96 ± 0.017 g/100 g FW) in the RA–BL sample, indicating that the color retention reagent/blanching combination is beneficial for controlling the loss of WSP.

*Lentinus edodes* are rich in VC, an antioxidant, and highly beneficial to the human body. However, several studies have reported that the nonenzymatic browning reaction is also associated with the VC content (Gu et al., 2018; Yang et al., 2021). During the processing of vegetables or fruits, VC is oxidized to carbonyl‐containing compounds, which then react or polymerize with other substances (Aghdam et al., 2020; Gu et al., 2018). The degradation reaction of VC has been identified as the main nonenzymatic browning reaction pathway that is responsible for browning in the food industry. (Paravisini & Peterson, 2019; Yang et al., 2021). In this study, the VC content was determined using different color protection conditions. The VC content of the RA sample was 3.54 ± 0.11 g/100 g FW, whereas that of the Con sample was 3.28 ± 0.15 g/100 g FW (*p* < .05; Figure 5b), with a slight increase in the VC content likely because of the use of *d*‐sodium erythorbate as the color protection reagent. The VC content of the RA–BL sample was 2.54 ± 0.08 g/100 g FW, nearly 2.6 times higher than that of the BL sample (0.99 ± 0.12 g/100 g FW; *p* < .05). Blanching destroys the VC structure and reduces its content, whereas the color protection method protects it from high‐temperature damage. In fact, in using the color protection method, a small fraction of the VC content in *L. edodes* was reduced, which in turn inhibited the nonenzymatic browning while retaining most of the VC content, thus preventing any nutrient loss.

## 4 CONCLUSIONS

Effectively inhibiting the browning effect is of great significance for the later stages of mushroom processing. In this study, two color protection methods (color retention reagent soaking and blanching) were explored to inhibit the browning of *L. edodes*. The effect of a composite color retention reagent was better than that of a single reagent, with the ΔE being the lowest when the composite color retention agent ratio was as follows: phytic acid = 0.1%, sodium citrate = 0.8%, and *d*‐sodium erythorbate = 0.5%. Specific blanching conditions (temperature = 90°C, period = 180 s) can also effectively inhibit the activity of PPO, which is the main oxidase responsible for the enzymatic browning of *L. edodes*. Furthermore, the effect of combining a color retention reagent and blanching on browning inhibition was studied. The oxidase activity was significantly reduced, with those of PPO = 5.88 ± 0.22 U/g FW and POD = 5.61 ± 0.78 U/g FW (control = 35.34 ± 2.02 U/g FW and 85.35 ± 7.18 U/g FW, respectively); however, the ΔE was only 0.38 ± 0.03 (control = 10.16 ± 0.17). The WSP content of the RA–BL sample was only slightly reduced (nearly5.9%), and the VC content of this sample was 2.54 g/100 g FW. Thus, using a compound color protectant in addition to blanching may effectively inhibit the browning and reduce the nutrient loss of *L. edodes*.

## CONFLICT OF INTEREST

The authors declare no conflict of interest.

## AUTHOR CONTRIBUTIONS

**Tong Lin:** Conceptualization (lead); Methodology (lead); Software (equal); Writing – original draft (lead). **Zhiguo Zhou:** Formal analysis (equal); Investigation (equal). **Chunmiao Xing:** Formal analysis (equal); Investigation (equal). **Jiahui Zhou:** Software (equal); Validation (equal). **Chunyan Xie:** Funding acquisition (lead); Project administration (lead); Resources (lead); Writing – review & editing (lead).

## INFORMED CONSENT

Written informed consent was obtained from all study participants.

## Supporting information

Supplementary Material

## Data Availability

The data that support the findings of this study are available from the corresponding author, upon reasonable request.
