# Supplementary material for: Effect of color protection treatment on the browning and enzyme activity of Lentinus edodes during processing
Source: Food Sci Nutr. 2022 Apr 20;10(9):2989–98. doi: 10.1002/fsn3.2895 (PMC9469847; doi:10.1002/fsn3.2895)
Supplement: Supplementary file 1 — Supplementary Material [file FSN3-10-2989-s001.docx]

**Supporting Information (SI)**

**Effect of** **color protection treatment on the browning and enzyme activity of** ***Lentinus edodes* during processing**

Tong Lin^a,b,c^, Zhiguo Zhou^a,b,c^, Chunmiao Xing^a^, Jiahui Zhou^a^, Gongjian Fan^d^ , and Chunyan Xie^a,^^b,c^ *

^a^ College of Life Science, Langfang Normal University, Langfang 065000, Hebei, China

^b^ Technical Innovation Center for Utilization of Edible and Medicinal Fungi in Hebei Province, Langfang 065000, Hebei, China

^c^Edible and Medicinal Fungi Research and Development Center of Hebei Universities, Langfang 065000, Hebei, China

^d^College of Light Industry and Food Engineering, Nanjing Forestry University, Nanjing 210037, China P.R., China

* Corresponding author: Chunyan Xie (Email: [1231597@lfnu.edu.cn](mailto:1231597@lfnu.edu.cn))

**Table S1.** Experimental range and levels of independent factors

| Coded level | Factor | | |
| --- | --- | --- | --- |
|  | A (phytic acid /%) | B (sodium citrate /%) | C (d-sodium erythorbate /%) |
| -1 | 0.09 | 0.6 | 0.4 |
| 0 | 0.10 | 0.8 | 0.5 |
| 1 | 0.11 | 1.0 | 0.6 |


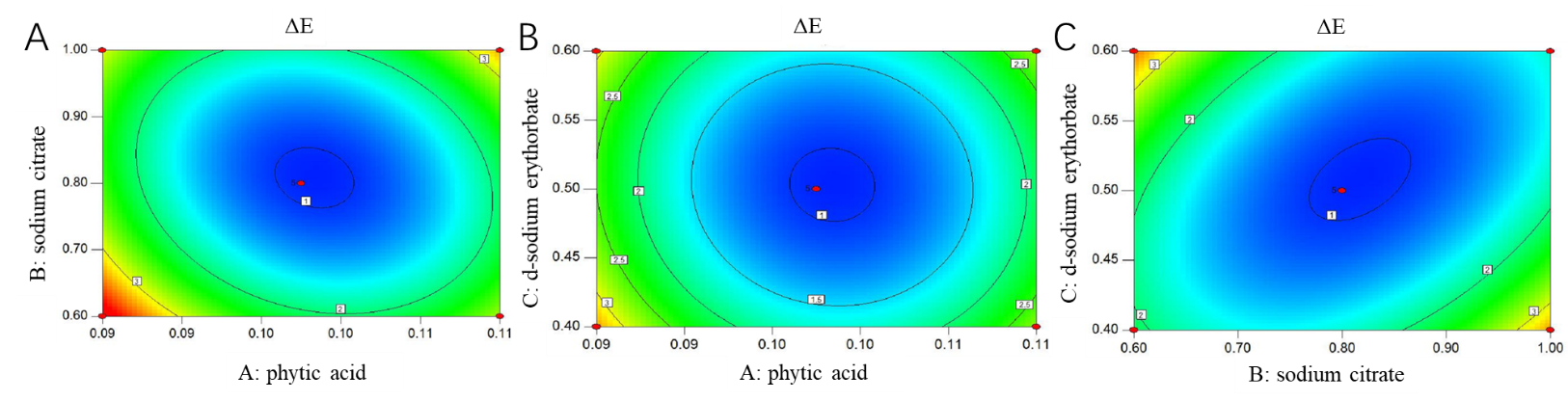


Fig S1. Contour plots showing the effect of phytic acid concentrations, sodium citrate concentrations and d-sodium erythorbate concentrations on total color difference by *L. edodes*. (A) Contour plots for the effect of phytic acid concentrations and sodium citrate concentrations. (B) Contour plots for the effect of phytic acid concentrations and d-sodium erythorbate concentrations. (C) Contour plots for the effect of sodium citrate concentrations and d-sodium erythorbate concentrations.
